# Bruceine D inhibits tumor growth and stem cell‐like traits of osteosarcoma through inhibition of STAT3 signaling pathway

**DOI:** 10.1002/cam4.2612

**Published:** 2019-10-21

**Authors:** Shangyu Wang, Hongzhi Hu, Binlong Zhong, Deyao Shi, Xiangcheng Qing, Cheng Cheng, Xiangyu Deng, Zhicai Zhang, Zengwu Shao

**Affiliations:** ^1^ Department of Orthopedics Union Hospital Tongji Medical College Huazhong University of Science and Technology Wuhan China

**Keywords:** bruceine D, cancer stem cells, osteosarcoma, STAT3 signaling pathway

## Abstract

Patients with osteosarcoma exhibiting resistance to chemotherapy or presenting with metastasis usually have a poor prognosis. Osteosarcoma stem cells (OSCs) are a potential cause of tumor metastasis, relapse, and chemotherapy resistance. Therefore, it is necessary to develop novel therapeutic drugs, which not only kill osteosarcoma cells but also target OSCs. This study aims to explore the anti‐osteosarcoma effects of Bruceine D (BD), a natural compound derived from *Brucea javanica*, and investigate its underlying mechanisms. Results demonstrated that BD could significantly inhibit cell proliferation and migration, induce cell cycle arrest, and promote apoptosis in osteosarcoma cells. Besides, BD could also suppress the sphere‐forming and self‐renewal ability of OSCs. Mechanistically, the inhibitory role of BD on osteosarcoma cell growth and migration including OSC stemness was partially executed through the inhibition of STAT3 signaling pathway. More importantly, BD showed significant anti‐osteosarcoma activity without obvious side effects in vivo. Collectively, the results of this study demonstrated that BD exerts a strong inhibitory effect on tumor growth and stem cell like traits of osteosarcoma which may be partially due to STAT3 inhibition, suggesting that BD maybe a promising therapeutic candidate against osteosarcoma.

## Graphical abstract

The present study provide evidence that BD has strong inhibitory effects on osteosarcoma stem cell like properties and tumor growth through repression of STAT3 signaling pathway, which suggests BD as a promising therapeutic agent for osteosarcoma.

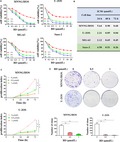

## 1 INTRODUCTION

Osteosarcoma, which occurs predominantly in children and adolescents, is the most common type of primary bone malignancy.^1^ With the advent of chemotherapy and advances in surgical technology, the 5‐year survival rates for patients with local osteosarcoma have significantly improved, attaining 70%.^1^, ^2^ Unfortunately, cure rate has not improved significantly over the past 30 years. Prognosis remains disappointing, especially for those who present with unresectable or metastatic tumors.^2^, ^3^ Therefore, it is necessary to seek novel drugs with more effective anti‐osteosarcoma activity and mild side effects.

Cancer stem cells (CSCs) are a small population of cells within the tumor that exhibit properties similar to normal stem cells but have tumor initiating ability in appropriate animal models.^4^, ^5^ Some research findings suggest that CSCs are responsible for tumor initiation, self‐renewal, propagation, and metastasis.^5^, ^6^, ^7^, ^8^ Since the CSC hypothesis was proposed, many studies have been conducted to identify osteosarcoma stem cells (OSCs).^7^ OSCs are reported to contribute to tumor relapse, metastasis, and resistance to chemotherapy. Current chemo‐drugs for the treatment of osteosarcoma could kill most proliferating tumor cells and reduce tumor size but have little effect on OSCs.^9^, ^10^, ^11^ Therefore, developing new drugs that affect both OSCs and non‐OSCs is warranted for osteosarcoma treatment.

Over the past decades, extracts from natural sources have received extensive attention due to their superiority in terms of safety, efficacy, availability, and multiple biological activities.^12^, ^13^ Bruceine D (BD) is a natural compound derived from the Chinese medicinal plant *Brucea javanica*.^14^ Several studies have shown that BD exhibits numerous pharmacological traits including anti‐inflammatory,^15^ anti‐parasitic,^16^ and hypoglycemic activities.^17^ Furthermore, BD exhibits significant anti‐tumor activity in pancreatic cancer,^18^ human chronic myeloid leukemia,^19^ and hepatocellular carcinoma.^20^ However, the underlying mechanism of action of BD on osteosarcoma remains unclear. Therefore, this study was designed to explore the role of BD on osteosarcoma in vitro and in vivo, to evaluate the underlying mechanism, and demonstrate its potential as a therapeutic candidate for osteosarcoma treatment.

## 2 MATERIALS AND METHODS

### 2.1 Cell culture and reagents

MNNG/HOS, U‐2OS, MG‐63, and Saos‐2 were obtained from Cell Bank of Shanghai Institute of Biochemistry and Cell Biology, Chinese Academy of Sciences (Shanghai, China). MNNG/HOS and MG‐63 cells were grown in α‐MEM (Hyclone), while Saos‐2 and U‐2OS were cultured in RPMI 1640 medium (Gibco) containing 10% FBS (Gibco) and 1% antibiotics (Penicillin and streptomycin). All the cells were grown at 37°C in a humidified incubator containing 5% CO2.

BD obtained from Shanghai Yuanye Bio‐Technology Co., Ltd (Shanghai, China), was dissolved in DMSO (Sigma‐ Aldrich) and stored at −20°C as single‐use aliquots. BCA protein assay kit was obtained from Beyotime. Matrigel was obtained from BD Bioscience. Verapamil and STAT3 inhibitor Stattic were obtained from Selleck. Primary antibodies against N‐cadherin, GAPDH, *β*‐actin, and cyclin D1 were purchased from Cell Signaling Technology. Antibodies against MMP‐9, MMP‐2, SHP1, STAT3, phospho‐STAT3 (Y105), JAK2, phospho‐JAK2 (Y1007, Y1008), CD133, Ki‐67, and *β*‐catenin were purchased from Abcam. Antibodies against Caspase 3, Bcl‐2, CDK2, CDK4, cyclin E, SOX‐2, OCT‐4, and Nanog were purchased from Proteintech.

### 2.2 Cell viability assay

Cells were seeded in 96‐well plates (3000 cells/well) and allowed to grow overnight. Cells were then treated with increasing concentrations of BD and cultured for further 24, 48, or 72 hours. The medium then was removed, and 100 μL CCK8 solution (Dojindo) was added to each well and incubated in the dark at 37°C for 2 hours. The absorbance at 450 nm was detected and normalized to the absorbance of the medium without cells. Cell viability of 0 μmol/L treatment groups was considered as 100%. IC50 values were calculated using Graphpad Prism 6.0.

### 2.3 Cell proliferation analysis

Carboxyfluorescein Succinimidyl Ester (CFSE) dilution assay was employed to detect cell proliferation as described previously.^21^ Osteosarcoma cells were labeled by CFSE using the CellTrace CFSE cell proliferation kit obtained from Thermo Fisher Scientific following the manufacturer's instructions and seeded into six‐well plates. After being cultured overnight, the labeled cells were stimulated with BD for 24, 48, or 72 hours, and cell proliferation index was detected and analyzed by flow cytometry (BD Biosciences).

### 2.4 Flow cytometry analysis for cell cycle distribution and apoptosis

Osteosarcoma cells were inoculated into six‐well plates and cultured overnight. After incubation with BD for 48 hours, cells were digested with trypsin and harvested. For cell cycle distribution analysis, the medium was removed, and cells were washed and digested with trypsin. Then, the harvested cells were fixed with 70% cold ethyl alcohol for at least 2 hours, washed with PBS, and incubated with propidium iodide according to manufacturer's protocols from cell cycle analysis kit (Beyotime). For analysis of apoptosis, both the adherent cells and suspension cells were collected, washed with PBS, and stained with Annexin V‐PE/7‐AAD (BD Biosciences). Cell cycle distribution and cell death was detected and analyzed by flow cytometry.

### 2.5 Western blotting

After stimulation with BD for 48 hours, cells were lysed using cold RIPA buffer and centrifuged at 4°C. Protein concentration of the collected supernatant was detected by the BCA Protein Assay Kit. Equal amounts (20‐40 μg) of total protein was separated by SDS‐PAGE (8%‐12%) and transferred to the 0.45 μm PVDF membrane (Millipore, Billerica, MA, USA). The membranes were blocked and incubated with corresponding primary antibody at 4°C for 8 hours, followed by incubation with peroxidase‐conjugated secondary antibody for 1 hour at room temperature. Protein expression was visualized by ECL detection reagents (Beyotime).

### 2.6 Colony formation assay

Osteosarcoma cells (500 cells/ well) were inoculated into six‐well plates and cultured overnight. These cells received BD treatment for 48 hours and were routinely cultured for another 6 days. Cell colonies were fixed with 4% paraformaldehyde and then stained with 0.1% crystal violet.

### 2.7 Sphere‐forming assay

Sphere‐forming assay was performed accordingly as described previously.^5^, ^11^ MNNG/HOS cells (2000 cells/well) were inoculated in six‐well ultralow attachment plates (Corning Inc) in serum‐free *α*‐MEM containing 1% B27 Supplement (Invitrogen), 10 ng/mL human EGF (Pepro Tech), and 10 ng/mL human bFGF (Pepro Tech). After 1 week, spheres were observed and photographed through a microscope, and sphere quantity was determined manually.

### 2.8 Analysis of side population (SP) and CD133 expression

MNNG/HOS cells were treated with 1 μmol/L BD for 48 hour. For SP cells analysis, cells were pre‐treated with or without 100 μmol/L Verapamil for 10 minutes before Hochest 33 342 staining. Then cells were incubated with Hochest 33 342 (5 μg/mL) in MEM containing 2% FBS for 70 minutes at 37°C with intermittent shaking.^22^ For analysis of surface marker CD133, cells were incubated with unconjugated rabbit anti‐CD133 monoclonal antibodies (1:100) for 1 hour at 4°C, followed by incubation with FITC‐ conjugated goat anti‐rabbit secondary antibodies for another 1 hour. Cells staining with secondary antibodies only were used as negative controls. After labelling, cells were washed twice with cold PBS and re‐suspended in cold PBS for flow cytometry analysis.

### 2.9 Wound healing assay

Osteosarcoma cells (About 3 × 10^5^ cells/well) were inoculated in six‐well plates and grown to full confluence. Cell monolayers were carefully scratched by a 200 μL pipette tip. Images of the scratch were observed and captured. Then, the scratched cells were routinely cultured to migrate for 24 hours in 1% FBS medium containing different concentrations of BD. After being washed with PBS, the wound gap was photographed, and the rate of wound healing was analyzed with the software Image J.

### 2.10 Transwell assay

Transwell system (Corning Costar) was applied to investigate the migration and invasion ability of osteosarcoma cells. Briefly, the chambers were pre‐coated without (migration) or with Matrigel (invasion). Hereafter, 3 × 10^4^ cells (migration assay) or 10 × 10^4^ cells (invasion assay) in 150 μL *α*‐MEM with or without indicated concentrations of BD were seeded onto the upper chamber and. 600 μL α‐MEM supplemented with 10% FBS was added to the lower compartments. After culturing for 20 hours (MNNG/HOS) or 48 hours (U‐2OS) at 37°C, the chambers were gently washed, fixed with 4% paraformaldehyde and stained with 0.1% crystal violet. Images of at least six randomly picked fields were photographed using a computer‐assisted microscope (Olympus) and the migration or invasion of cells was quantitated manually.

### 2.11 Xenograft tumor model

Four‐week‐old female BALB/c nude mice were obtained from Changzhou Cavens Laboratory Animal Technology Co. Ltd. and housed in specific pathogen‐free conditions. For in vivo study, about 3 × 10^6^ MNNG/HOS cells suspended in 100 μL PBS were subcutaneously inoculated into the right flank of the nude mice. Three days after injection, the mice were randomly assigned into different groups (Control, BD 2.5 mg/kg, BD 5 mg/kg, Cisplatin). BD (2.5 or 5 mg/kg) in 100 μL saline was injected intraperitoneally every alternate day. The control group received equal amounts of saline. As a positive control group, Cisplatin (2 mg/kg) was injected intraperitoneally twice a week. The length and width of the tumors, and body weights of each mouse were measured every alternate day. Tumor volumes were calculated by the formula *V* = 1/2 (width^2^ × length). At the end of the experiment, tumors were carefully removed and weighed. After fixation, mice organs were embedded in paraffin, cut into slices, and subjected to hematoxylin and eosin (H&E) staining. The animal experiments were approved by the Animal Care and Ethics Committee of Huazhong University of Science and Technology (IACUC Number: S848).

### 2.12 Immunohistochemistry analysis

Paraformaldehyde fixed tumor tissues were embedded in paraffin and sliced by a microtome. After being deparaffinized and rehydrated along with antigen retrieval, the tumor slices were blocked and stained with antibodies against Ki‐67 (1:200), p‐STAT3 (1:100), MMP‐2 (1:100), and MMP‐9 (1:100). Images of the sections were photographed using a light microscope.

### 2.13 Statistics analysis

Unpaired Student's *t* test was used to analyze the differences between the two groups in vitro and in vivo. GraphPad Prism 6.0 was used for statistical analysis. Differences among groups were considered statistically significant at *P < .05, **P < .01. Quantitative data were expressed as mean ± SD for at least three independent experiments.

## 3 RESULTS

### 3.1 BD inhibits proliferation of osteosarcoma cells

**Figure 1:**
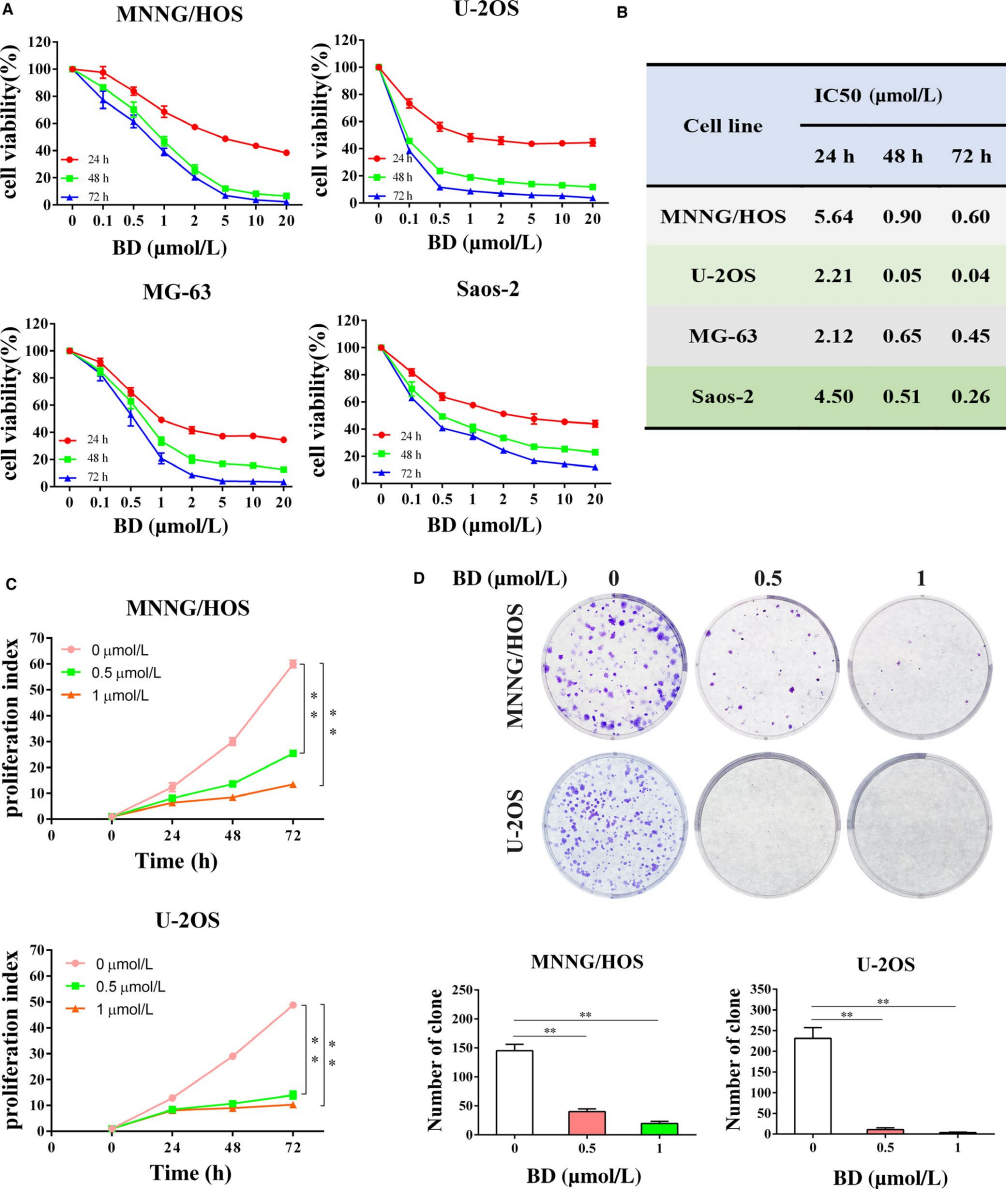
Effects of BD on osteosarcoma cell proliferation. (A and B) Four different human osteosarcoma cell lines (MNNG/HOS, U‐2OS, MG‐63, and Saos‐2) were treated with increasing concentrations of BD for 24, 48, and 72 h, respectively. Cell viability was measured using a CCK8 assay, and IC50 values were calculated and shown. (C) MNNG/HOS and U‐2OS cells were labeled with CFSE and treated with BD for 24, 48, and 72 h. Cell proliferation was evaluated using Flow Cytometer. (D) Osteosarcoma cells were treated with BD for 48 h and cultured for another 6 d. Cell colonies were fixed, stained, and counted. (Quantitative data were expressed as mean ± SD from at least three independent experiments. *P < .05, **P < .01, BD treatment vs control)

Primarily, the role of BD on osteosarcoma cell viability was assessed by CCK8 assay in MNNG/HOS, U‐2OS, MG‐63, and Saos‐2 after being exposed to different concentrations of BD for 24 to 72 hours, respectively. Results showed that BD exhibited a time and dose dependent inhibitory effect on osteosarcoma cells (Figure 1A). The IC50 values of BD for each cell lines at different time points were calculated (Figure 1B). A CFSE dilution assay was also conducted to determine the effects of BD on osteosarcoma cell proliferation. The data indicated that BD remarkably suppressed osteosarcoma cell proliferation (Figure 1C). Anti‐proliferative effect of BD on osteosarcoma cells was further verified with colony‐formation assay. Stimulation with BD significantly reduced the number and size of colonies (Figure 1D). Conclusively, these findings indicate that BD could significantly suppress osteosarcoma cell proliferation in vitro.

### 3.2 BD inhibits cell cycle progression, and promotes apoptosis in osteosarcoma cells

**Figure 2:**
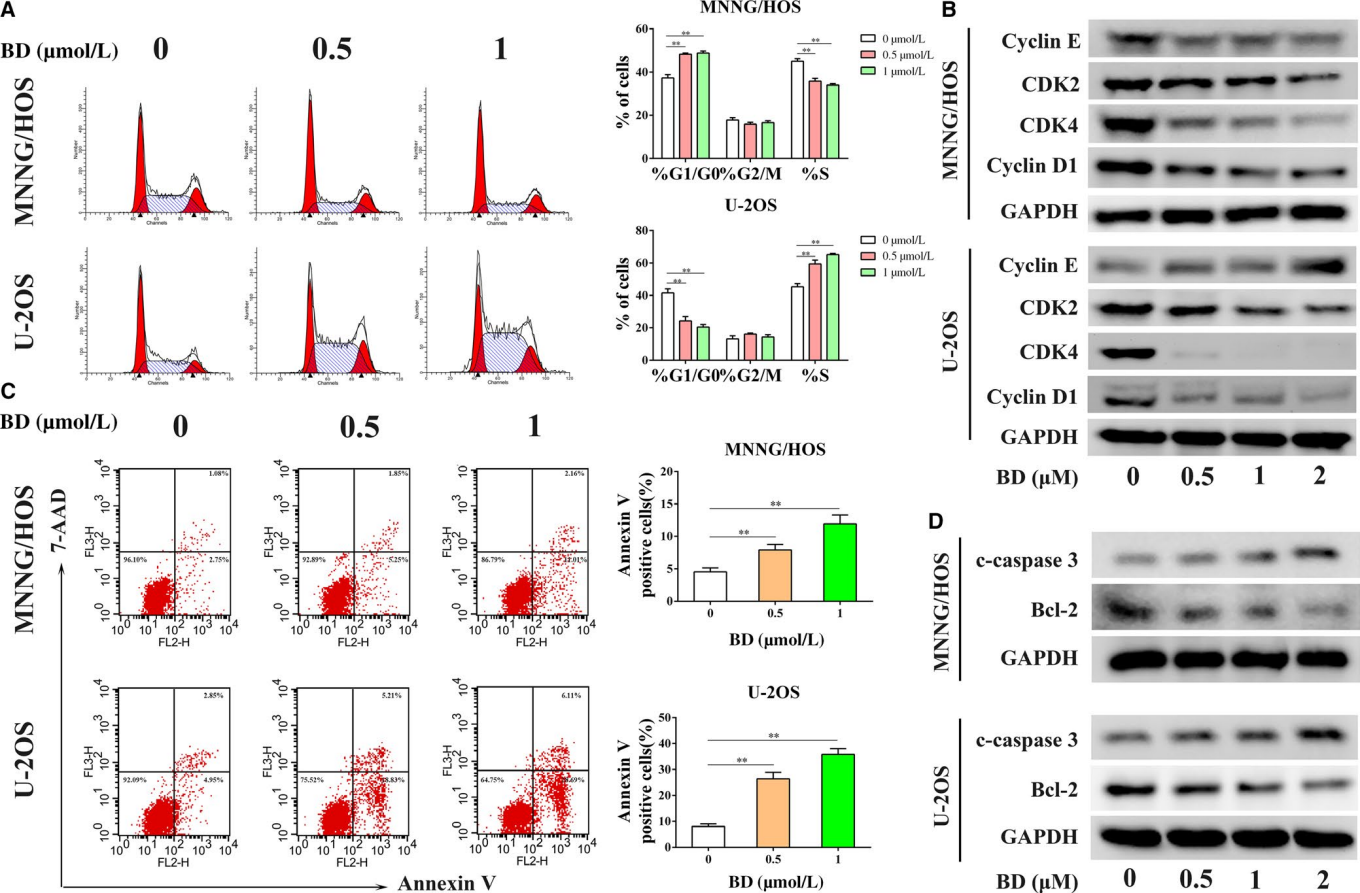
Effects of BD on cell cycle distribution and apoptosis in osteosarcoma cells. (A) Representative cell cycle distribution and statistical analyses of MNNG/HOS and U‐2OS cells treated with different concentrations of BD for 48 h. (B) The expression level of some cell cycle‐regulated proteins (CDK2, CDK4, Cyclin D1, and Cyclin E) were measured by western blot after treatment with BD for 48 h. (C) Cells were treated with different concentrations of BD for 48 h. Apoptosis was determined using flow cytometry after staining with Annexin V‐PE/7‐AAD. (D) The expression levels of two apoptosis‐related proteins (cleaved caspase 3 and Bcl‐2) were measured by western blot after BD treatment for 48 h. (Quantitative data were expressed as mean ± SD from at least three independent experiments. **P < .01, BD treatment vs control)

To investigate whether the anti‐proliferative activity of BD was related to cell cycle arrest, cell cycle distribution was evaluated after BD stimulation in MNNG/HOS and U‐2OS cells. The number of cells in G0/G1 phase was significantly increased, accompanied by a significant reduction of cells in S phase in MNNG/HOS cells after BD treatment (Figure 2A). However, in U‐2OS, BD treatment led to accumulation of cells in S phase and a reduction of cells in the G0/G1 phase (Figure 2A). To further explore the potential mechanism, we detected the expression of several cell cycle regulatory proteins. The expression of Cyclin D1, CDK4 and CDK2 was notably downregulated after BD treatment in both MNNG/HOS and U‐2OS cells. However, the expression of Cyclin E was downregulated in MNNG/HOS cells and upregulated in U‐2OS cells (Figure 2B). These results indicate that BD induces cell cycle arrest by changing the expression of essential regulatory proteins in cell cycle progression.

To explore whether the BD‐induced reduction in osteosarcoma cell viability was also related to apoptosis, we analyzed the apoptotic rate of osteosarcoma cells after BD stimulation by Annexin V‐PE/7‐AAD double staining. BD treatment induced apoptosis in both MNNG/HOS and U‐2OS cells (Figure 2C). Furthermore, we also detected the levels of apoptosis‐related proteins. BD treatment notably increased the expression of proapoptotic protein cleaved caspase 3, and correspondingly reduced the level of antiapoptotic protein Bcl‐2 in vitro (Figure 2D).

Conclusively, all these findings demonstrate that the inhibitory role of BD on osteosarcoma cell viability appears to be mediated through induction of cell cycle arrest and apoptosis.

### 3.3 BD suppresses osteosarcoma cell migration and invasion in vitro

**Figure 3:**
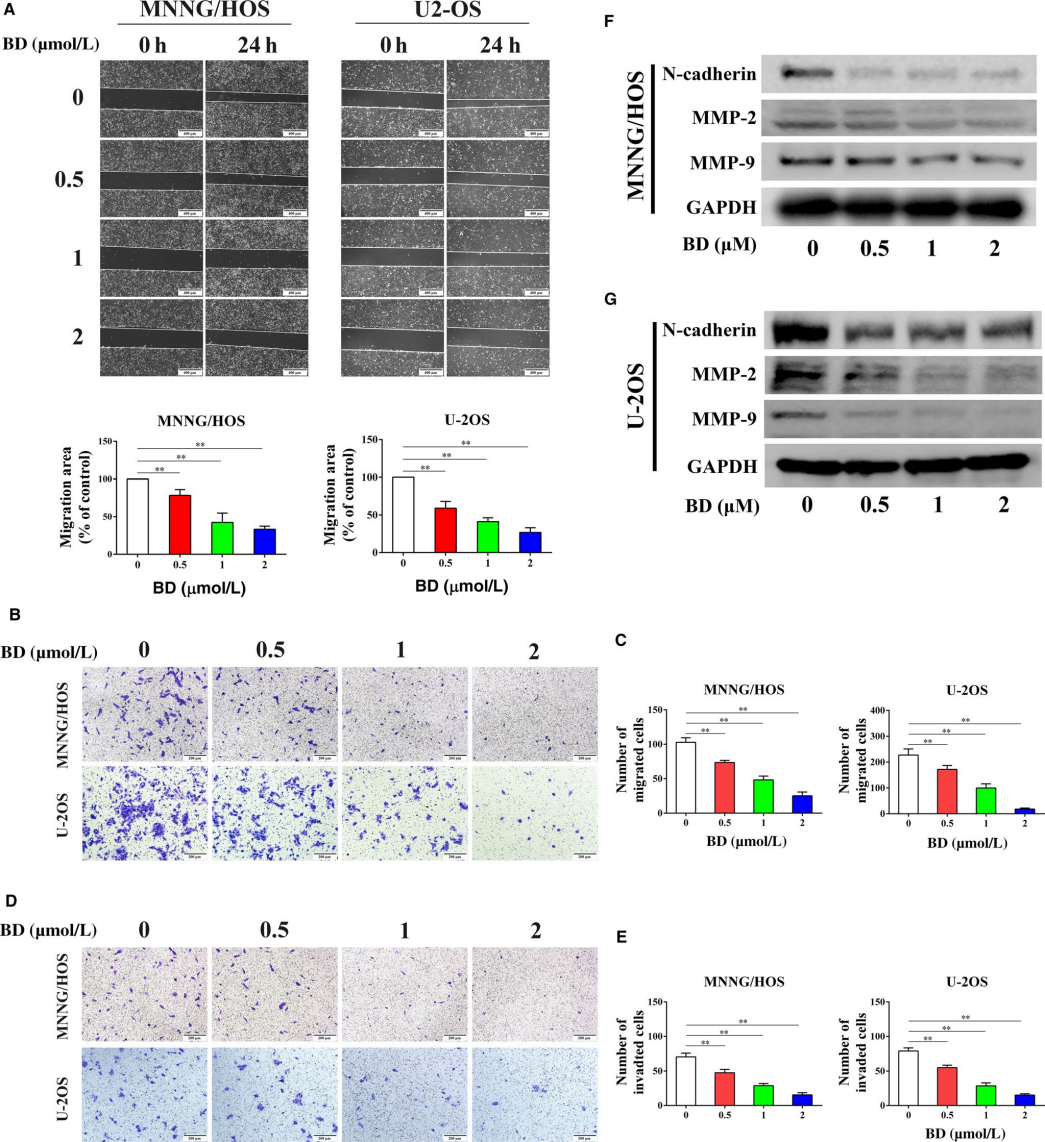
Effects of BD treatment on osteosarcoma cell migration and invasion. (A) MNNG/HOS and U‐2OS cells were plated, scratched and incubated with different concentrations of BD for 24 h. Wound healing rates were measured by manual counting. Scale bar, 400 μm. Representative images of Transwell migration (B and C) and invasion (D and E) assays in MNNG/HOS and U‐2OS are shown. The migrated or invaded cells were quantified manually. Scale bar, 200 μm. (F and G) MNNG/HOS and U‐2OS cells were treated with different concentrations of BD for 48 h. EMT‐related markers (N‐cadherin, MMP‐2, and MMP‐9) were evaluated by Western blot. (Quantitative data was expressed as mean ± SD from at least three independent experiments. **P < .01, BD treatment vs control)

Wound healing assays and Transwell assays were conducted to evaluate the effects of BD on the osteosarcoma cell migration and invasion. The wound healing rate was significantly impaired after treatment with different concentrations of BD for 24 hours (Figure 3A). The anti‐migration effect of BD on osteosarcoma cells was further verified by Transwell migration assay without Matrigel (Figure 3B,C). In addition, BD treatment could also significantly suppress the invasive ability of osteosarcoma cells (Figure 3D,E). N‐cadherin and MMPs play important role in tumor migration and metastasis. Therefore, we measured the expression of N‐cadherin, MMP‐9 and MMP‐2 in osteosarcoma cells after BD stimulation. As shown in Figure 3F,G, BD treatments notably reduced the protein levels of these epithelial‐mesenchymal transition (EMT) markers.

### 3.4 BD inhibits osteosarcoma cell proliferation and migration through inhibition of STAT3 signaling pathway

**Figure 4:**
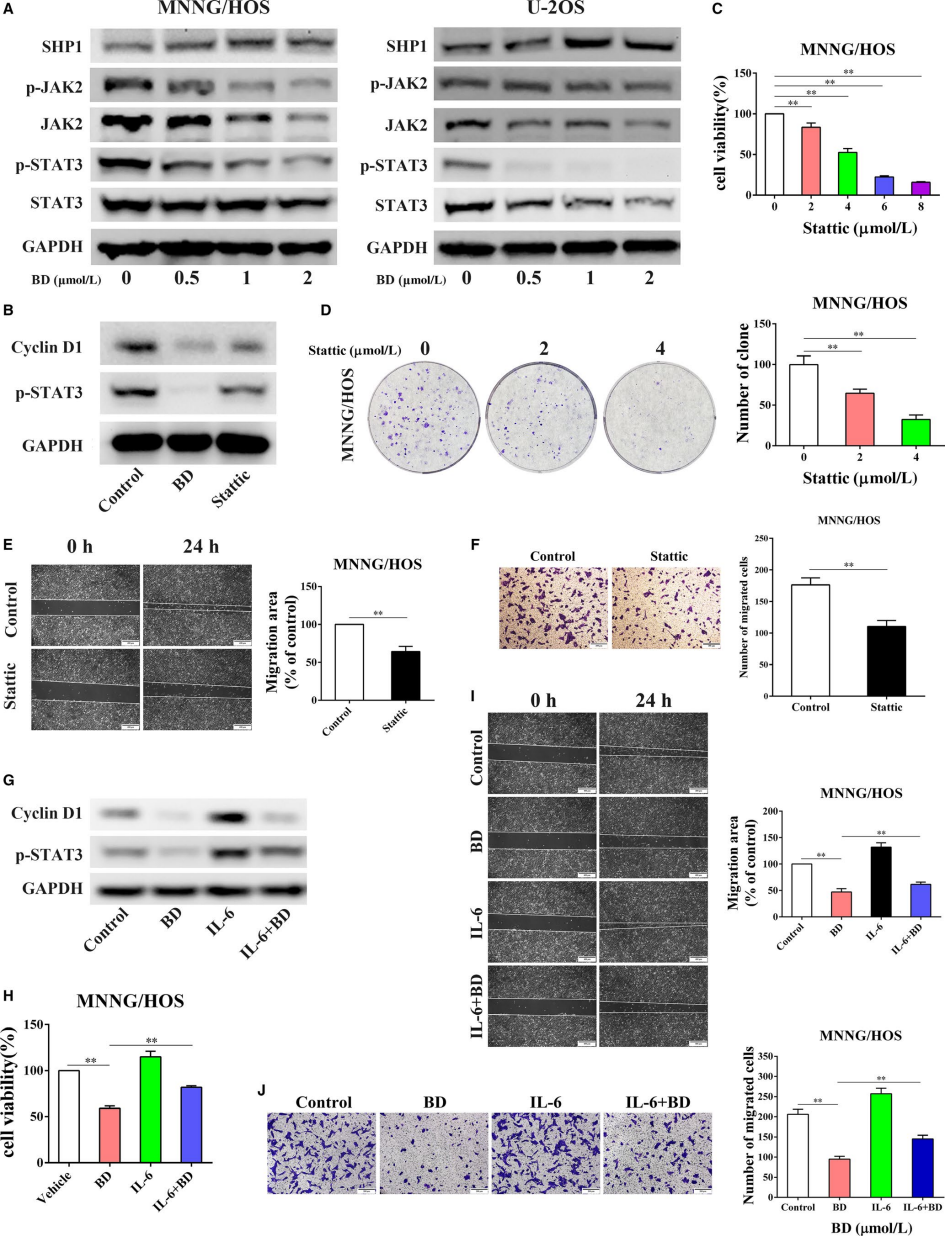
BD inhibits osteosarcoma proliferation and migration through inhibition of the STAT3 signaling pathway. (A) MNNG/HOS and U‐2OS cells treated with different concentrations of BD for 48 h. The expression of the critical proteins (SHP1, JAK2/p‐JAK2, and STAT3/p‐STAT3) involved in STAT3 signaling pathway was examined. (B) STAT3 signaling inhibitor Stattic could inhibit the activation of STAT3 and its target gene, Cyclin D1, in MNNG/HOS cells. (C) After being treated with increasing concentrations of Stattic, cell viability was examined by CCK8 assay in MNNG/HOS cells. (D) Stattic significantly reduced colony formation of MNNG/HOS cells. The effects of Stattic on MNNG/HOS cell migration was evaluated by wound healing assay (E) and Transwell assay (F). (G) IL‐6 could effectively activate STAT3 signaling and partly rescue the suppression of BD treatment in MNNG/HOS cells. The impairment of BD on cell proliferation (H), wound healing (I), and Transwell migration (J) was partly rescued by IL‐6 in MNNG/HOS cells. (Quantitative data were expressed as mean ± SD from at least three independent experiments. **P < .01, BD treatment vs control, BD treatment vs IL‐6 + BD treatment)

STAT3 activation and its oncogenic role have been demonstrated in several malignancies including osteosarcoma.^23^, ^24^, ^25^ Therefore, to investigate whether STAT3 pathway was related to the anti‐osteosarcoma activity of BD, we investigated the role of BD on the activation of STAT3 signaling pathway. Figure 4A showed that the expression of phosphorylated JAK2 (Y1007 + Y1008)/STAT3 (Y705) was notably decreased both in MNNG/HOS and U‐2OS cells after BD treatment. Furthermore, the nonreceptor protein tyrosine phosphatase SHP1 (SH2 domain containing phosphatase 1), which has been reported to negatively regulate STAT3 phosphorylation,^26^, ^27^ was upregulated upon treatment with BD (Figure 4A). These results demonstrate that BD stimulation effectively represses the activation of STAT3 signaling pathway.

To further confirm whether BD mediates the impeding effects on cell growth and migration by inhibiting STAT3 signaling pathway, we investigated the effects of Stattic, a potent inhibitor of STAT3 activation,^28^ on osteosarcoma cells viability and migration. Consistent with BD treatment, Stattic inhibited STAT3 phosphorylation and its downstream target genes, such as cyclin D1 (Figure 4B). CCK8 assay, wound healing and Transwell assay demonstrated that inhibition of STAT3 suppressed cell viability and migration ability (Figure 4C‐F). Furthermore, activating STAT3 by IL‐6 (Figure 4G), a commonly used STAT3 activator,^29^ could partially rescue the negative effects of BD on cell proliferation (Figure 4H) and migration (Figure 4I,J). Therefore, these findings demonstrate that STAT3 deactivation was implicated in BD‐induced inhibition of osteosarcoma growth and migration, at least partially.

### 3.5 BD inhibits the stem cell like traits of osteosarcoma cells in vitro

**Figure 5:**
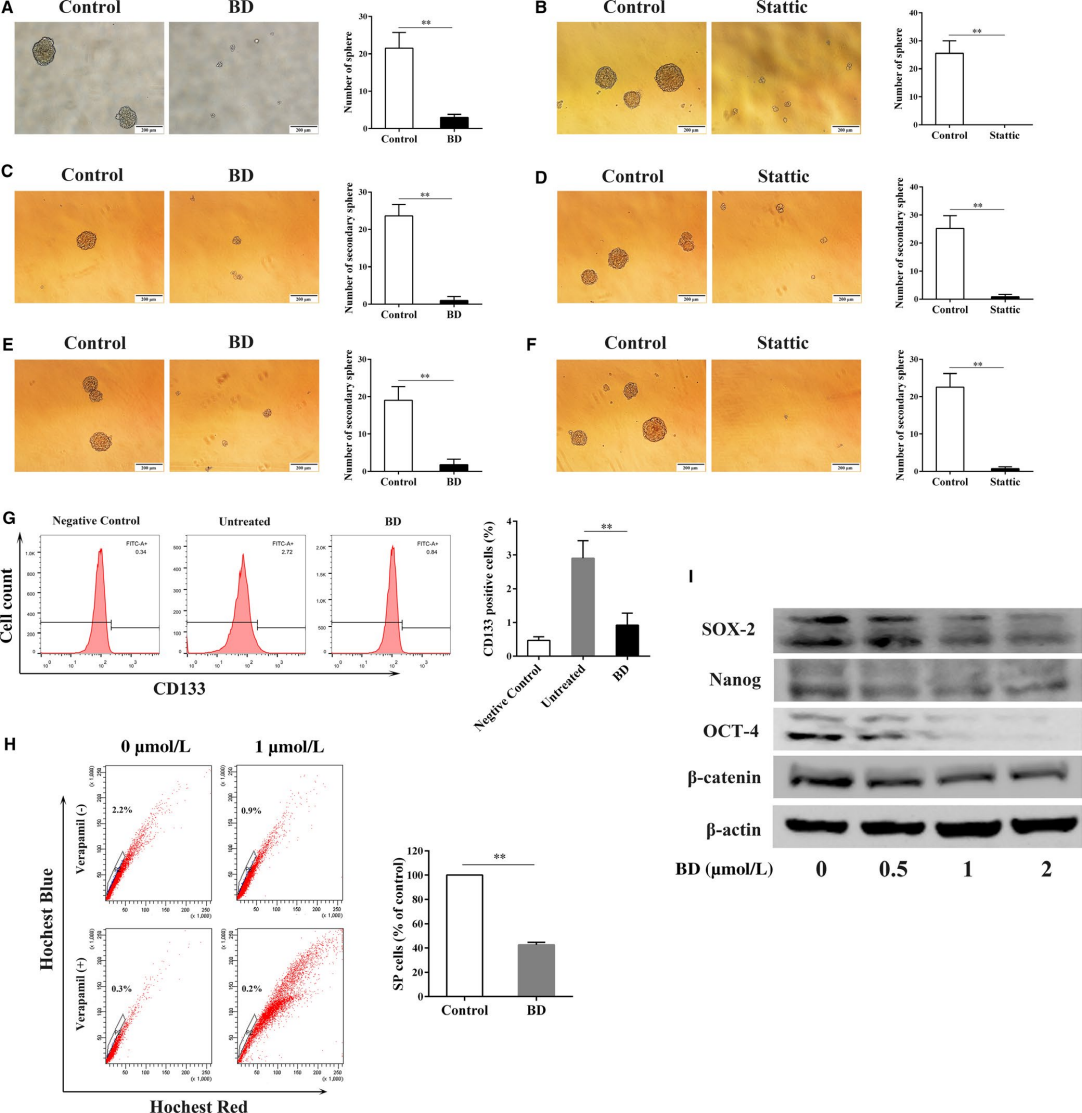
BD inhibits stem cell like properties of osteosarcoma cells. The sphere‐forming capacity of MNNG/HOS cells was evaluated after treatment with 1 μmol/L BD (A) or 4 μmol/L Stattic (B). Scale bar, 200 μm. Primary spheres treated with indicated drugs were disassociated into single cells and the secondary sphere formation capacity was analyzed without further treatment of BD (C) or Stattic (D). Scale bar, 200 μm. Primary spheres without BD (or Stattic) treatment were disassociated into single cells and the secondary sphere formation capacity was evaluated after treatment with 1 μmol/L BD (E) or 4 μmol/L Stattic (F). Scale bar, 200 μm. MNNG/HOS cells were treated with 1 μmol/L BD for 48 h and harvested. Then the cells were stained with anti‐CD133 antibody to detect the proportion of CD133 positive cells (G), or incubated with Hochest 33 342 for SP fraction analysis (H). (I) Expression of β‐catenin and stem cell markers (SOX‐2,OCT‐4, and Nanong) in MNNG/HOS cells treated with or witout BD for 48 h was detected by western blot. (Quantitative data were expressed as mean ± SD from at least three independent experiments. **P < .01 vs control)

CSCs, a small subpopulation of cells within a tumor, are considered to possess the capacity to self‐renew and maintain the tumor phenotype,^6^, ^10^ which may annul the clinical treatment.^7^, ^30^ Here we investigated whether BD could inhibit sphere‐forming ability of osteosarcoma cells and self‐renewal capacity of OSCs using sphere‐forming assay.^5^, ^31^ Osteosarcoma cells treated with BD formed much smaller and significantly fewer spheres (Figure 5A). Furthermore, secondary sphere‐forming assay was performed to test whether BD affected the self‐renewal ability of OSCs. Results showed that BD treatment in first generation spheres significantly reduced the secondary sphere‐forming efficiency without further BD treatment (Figure 5C). We also found that single cell suspension disassociated from non‐treated first‐generation spheres formed fewer and smaller secondary spheres (Figure 5E) when treated with BD. In addition, we performed flow cytometry to analyze CD133 positive cells and SP cells in MNNG/HOS cells, both of which were widely used for identifying CSCs. Results showed that BD treatment significantly reduced the proportion of CD133 positive cells and SP cells (Figure 5G,H). These results suggest that BD treatment reduced the proportion of stem‐like osteosarcoma cells and impaired the self‐renewal ability of OSCs.

Furthermore, we investigated whether BD treatment influences the expression of several stem cell markers (SOX2, OCT‐4 and Nanog) in MNNG/HOS cells. Results showed that these stem cell markers were notably downregulated after BD treatment (Figure 5I). We also detected the effects of BD treatment on *β*‐catenin expression, which is reported to play pivotal roles in regulating stem cell properties.^32^ The data showed that BD stimulation reduced *β*‐catenin expression in MNNG/HOS cells (Figure 5I). It has been reported that STAT3 signaling repression was implicated in resveratrol induced elimination of OSCs.^33^ Our results consistently showed that inhibition of STAT3 signaling using Stattic, significantly inhibits sphere formation and self‐renewal ability of OSCs (Figure 5B,D,F).

In summary, BD inhibited stem cell like properties and negatively regulated the self‐renewal ability of OSCs, and these effects could be through the repression of STAT3 signaling pathway.

### 3.6 BD suppresses osteosarcoma tumor growth in vivo

**Figure 6:**
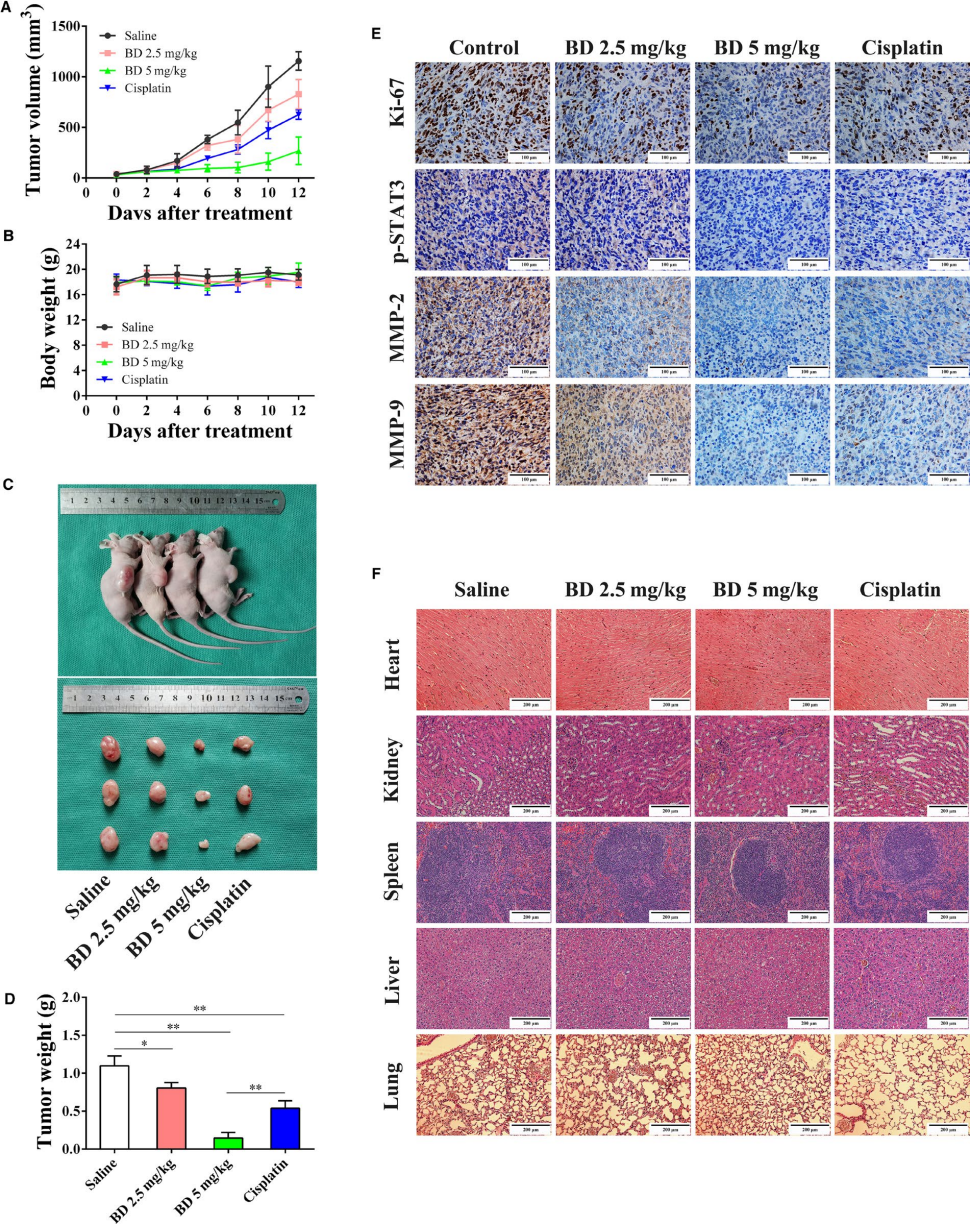
BD inhibits the growth of osteosarcoma in vivo. Nude mice bearing human osteosarcoma xenograft tumors were treated as described in Materials and Methods (A) The length and width of the tumors were measured at the indicated time points and the volumes were calculated. Data represent the mean ± SD of tumor volume from three mice. (B) The weight of each mouse was weighed every alternate day. The data are shown as the mean ± SD of 3 mice. (C) Representative images of the nude mice bearing osteosarcoma xenografts from different groups and the morphology of the xenografts are shown. (D) Tumor weights were measured and compared. The data represent the mean ± SD of the volume of tumors from three mice. (E) The expression of Ki‐67, p‐STAT3, MMP‐2, and MMP‐9 was evaluated via IHC in different groups, and representative images are shown. Scale bar, 100 μm. (F) Representative histological profiles of major organs in different treatment groups were detected by H&E staining. Scale bar, 100 μm. (Quantitative data were expressed as mean ± SD from three mice in each group. *P < .05, **P < .01 vs control, BD 5 mg/kg vs Cisplatin group)

In vitro studies indicated that BD exhibited obvious inhibitory effects on osteosarcoma cells. We further investigated the in vivo effects of BD by a xenograft model. The tumor size was markedly reduced in both BD and Cisplatin‐treated mice (Figure 6A,C). In accordance with tumor growth curve, tumor weights of mice in the BD treatment groups and Cisplatin group were also significantly decreased (Figure 6D). Furthermore, tumor sizes and tumor weights in mice treated with high‐dose BD (5 mg/kg) were significantly reduced than that of Cisplatin (Figure 6A,C,D), one of the first line chemo‐drugs for osteosarcoma treatment. During the experimental period, there were no obvious differences in body weights between the control and BD treated groups (Figure 6B), and data of H&E staining indicated that there were no discernable pathologic changes in major organs of mice in different groups (Figure 6F).

We also evaluated the expression of proliferative marker Ki‐67, to further confirm the inhibitory role of BD on osteosarcoma tumor growth. Ki‐67 positive cells in the tumors from mice receiving BD or Cisplatin were markedly reduced than that in the control group (Figure 6E). Consistent with tumor sizes and weights, the BD 5 mg/kg group showed even lower expression of Ki‐67 than the Cisplatin group (Figure 6E). To investigate the effects of BD on osteosarcoma invasive ability in vivo, we evaluated lung metastasis and detected the expression of MMP‐2 and MMP‐9 in different groups. Despite that no lung metastatic foci were observed in any of the groups, the expression level of MMP‐2 and MMP‐9 was markedly decreased in BD treated xenografts (Figure 6E). In addition, we assessed the effects of BD on STAT3 phosphorylation in vivo. Consistent with the in vitro data, BD treatment decreased the expression of p‐STAT3 in xenograft tumors (Figure 6E).

Taken together, these findings indicate that BD could significantly inhibit osteosarcoma tumor growth and metastatic potential in vivo without obvious organ toxicity.

## 4 DISCUSSION

The advent of adjuvant therapies and advances in surgical techniques, have caused the overall survival rates of osteosarcoma patients to reach 60%‐75%.^1^, ^2^, ^3^ However, the 5‐year survival rate for those with metastasis remains less than 30% and is almost unchanged over the past three decades.^2^, ^3^, ^5^ Current chemo‐drugs are limited due to their side effects and development of resistance.^34^ Therefore, identification of efficacious agents with less side effects is of paramount importance for osteosarcoma treatment. Inspired by the success of Artemisinin (Qinghaosu) in the clinic, many researchers have started investigating natural extracts.^35^ Some natural extracts from plant exert potent anticancer activity with low toxicity. Many studies have explored their anti‐tumor effects and regulating mechanisms.^35^, ^36^ In this study, we demonstrate that BD, a natural compound isolated from *B javanica* fruit,^14^ diminishes osteosarcoma tumor growth and suppresses stemness properties of osteosarcoma. More importantly, BD could suppress the in vivo growth of osteosarcoma without obvious toxicity. Mechanistically, the suppressive effects of BD on osteosarcoma could be executed through inhibition of STAT3 pathway. These findings suggest that BD could be a promising therapeutic candidate against osteosarcoma.

Anomaly in cell cycle progression underlies the unscheduled cell proliferation that characterizes cancer.^37^, ^38^, ^39^ Induction of cell cycle arrest is an important mechanism through which chemo‐drugs exert their anti‐cancer activities.^40^ Our results indicate that treatment of BD could induce cell cycle arrest and suppress proliferation of osteosarcoma cells. Cell cycle progression is controlled by a number of cyclin‐dependent kinases (CDK) and their regulatory partners, the cyclins.^39^ Cyclin D usually form complexes with CDK4 or CDK6, which play important roles in G1 phase progression.^41^ CDK2 could form complexes with cyclin E or cyclin A, and control G1‐S phase transition and S phase progression, respectively.^39^, ^42^ In this study, BD treatment induced G0/G1 phase arrest and notably reduced the expression of cyclin D1, CDK4, CDK2, and cyclin E in MNNG/HOS cells. However, BD treatment led to S phase arrest, in spite of the downregulation of cyclin D1, CDK4, and CDK2 expression, in U‐2OS cells. We found that BD stimulation upregulated the expression level of cyclin E in U‐2OS cells, which is reported to control cell cycle progression from G1 into S phase.^42^ Therefore, the upregulated cyclin E may have a compensatory role to drive U‐2OS cells progressing into S phase. Decreased expression of CDK2 has been reported in S‐phase arrest.^41^, ^43^ Thus, the decreased expression of CDK2 in U‐2OS cells may be another reason for S phase arrest. Many anti‐cancer drugs exert their anticancer activities by promoting apoptosis in cancer cells. We found that BD treatment induced significant apoptosis in osteosarcoma cells, as detected by Annexin V/7‐AAD staining, and expression of cleaved caspase 3 and Bcl‐2.

Constitutive activation of the STAT3 signal pathway has been reported to play an essential role in tumor cell growth, survival, and metastasis.^23^, ^24^, ^44^ Previous studies have shown that STAT3 activation contributes to tumor progression in many cancers, including osteosarcoma,^24^, ^44^, ^45^ and overexpression of phospho‐STAT3 was related to poor prognosis in osteosarcoma patients.^23^ In addition, another study has demonstrated that pharmacological inhibition of STAT3 exhibits significant anti‐osteosarcoma effects.^45^ In this study, we showed that BD significantly inhibits cell proliferation and migration, notably repressed the phosphorylation of JAK2 and STAT3 in osteosarcoma cells, and increased the protein level of SHP1, a negative regulator of STAT3 signaling pathway.^45^ We also found that inhibition of STAT3 signaling using Stattic^28^ significantly inhibited osteosarcoma cell growth and migration. Furthermore, activation of STAT3 by IL‐6 stimulation weakened the inhibitory effects of BD on cell growth and migration. Besides, IHC analysis of xenograft tumors revealed that BD treatment markedly decreased the expression of p‐STAT3, MMP‐2, and MMP‐9. These findings indicate that BD may exert its antitumor activity partially due to the inhibition of STAT3 signaling in osteosarcoma. However, the complete regulatory mechanism through which BD inhibits the activity of STAT3 signaling pathway still needs further evaluation.

Accumulating evidence has demonstrated that osteosarcoma possesses CSCs and these subpopulations are considered to be involved in chemo‐resistance, tumor metastasis and recurrence, which should be a promising target for developing novel drugs.^7^, ^31^, ^46^ Several methods have been developed to isolate/enrich subpopulation of cells with stem cell properties within osteosarcoma.^46^, ^47^, ^48^ In the present study, we used sphere‐forming assay, a commonly used strategy to isolate CSCs,^5^, ^11^, ^31^, ^49^ to enrich OSCs and examine the effects of BD on OSCs. Here, our results revealed that BD exhibited the capacity to inhibit the stem cell like traits of osteosarcoma cells and inhibit OSCs self‐renewal ability. Previous studies have reported that STAT3 activation was important in maintaining CSCs, and inhibition of STAT3 signaling may be involved in CSCs stemness attenuation.^33^, ^50^, ^51^ Consistent with these findings, we found that BD could deactivate STAT3 signaling and inhibition of STAT3 using Stattic significantly suppressed the sphere‐forming and self‐renewal capacity of osteosarcoma cells. Collectively, our data indicated that inhibitory effects of BD on OSC stemness may be through the suppression of STAT3 signaling, and BD could be a promising agent for OSC‐targeted therapy. However, the detailed regulatory role of STAT3 signaling in BD‐induced stemness attenuation of OSCs needs further evaluation.

In summary, our results demonstrate that BD is able to distinctly suppress osteosarcoma cell proliferation, migration, invasion and stem cell like properties in vitro. Furthermore, BD can also inhibit xenograft tumor growth in vivo without obvious side effects. The potential mechanism through which BD exhibits its anti‐osteosarcoma activity could be through the repression of STAT3 activation. All these findings indicate that BD could be included in potential therapeutic regimen for osteosarcoma treatment. However, further research is necessary for better understanding of the mechanism of BD on osteosarcoma.

## CONFLICT OF INTEREST

The authors declare that they have no competing interest.

## Data Availability

The data that support the findings of this study are available from the corresponding author upon reasonable request.

## References

[1] Bishop MW , Janeway KA , Gorlick R . Future directions in the treatment of osteosarcoma. Curr Opin Pediatr. 2016;28(1):26‐33. 26626558 10.1097/MOP.0000000000000298 PMC4761449

[2] Mirabello L , Troisi RJ , Savage SA . Osteosarcoma incidence and survival rates from 1973 to 2004: data from the surveillance, epidemiology, and end results program. Cancer. 2009;115(7):1531‐1543. 19197972 10.1002/cncr.24121 PMC2813207

[3] Huang K , Chen Y , Zhang R , et al. Honokiol induces apoptosis and autophagy via the ROS/ERK1/2 signaling pathway in human osteosarcoma cells in vitro and in vivo. Cell Death Dis. 2018;9(2):157. 29410403 10.1038/s41419-017-0166-5 PMC5833587

[4] Tang DG . Understanding cancer stem cell heterogeneity and plasticity. Cell Res. 2012;22(3):457‐472. 22357481 10.1038/cr.2012.13 PMC3292302

[5] Qu H , Xue Y , Lian W , et al. Melatonin inhibits osteosarcoma stem cells by suppressing SOX9‐mediated signaling. Life Sci. 2018;207:253‐264. 29689273 10.1016/j.lfs.2018.04.030

[6] Adorno‐Cruz V , Kibria G , Liu X , et al. Cancer stem cells: targeting the roots of cancer, seeds of metastasis, and sources of therapy resistance. Cancer Res. 2015;75(6):924‐929. 25604264 10.1158/0008-5472.CAN-14-3225 PMC4359955

[7] Siclari VA , Qin L . Targeting the osteosarcoma cancer stem cell. J Orthop Surg Res. 2010;5:78. 20979639 10.1186/1749-799X-5-78 PMC2988747

[8] Batlle E , Clevers H . Cancer stem cells revisited. Nat Med. 2017;23(10):1124‐1134. 28985214 10.1038/nm.4409

[9] Levings PP , McGarry SV , Currie TP , et al. Expression of an exogenous human Oct‐4 promoter identifies tumor‐initiating cells in osteosarcoma. Cancer Res. 2009;69(14):5648‐5655. 19584295 10.1158/0008-5472.CAN-08-3580 PMC2841219

[10] Adhikari AS , Agarwal N , Wood BM , et al. CD117 and Stro‐1 identify osteosarcoma tumor‐initiating cells associated with metastasis and drug resistance. Cancer Res. 2010;70(11):4602‐4612. 20460510 10.1158/0008-5472.CAN-09-3463 PMC3139225

[11] Tang QL , Zhao ZQ , Li JC , et al. Salinomycin inhibits osteosarcoma by targeting its tumor stem cells. Cancer Lett. 2011;311(1):113‐121. 21835542 10.1016/j.canlet.2011.07.016

[12] Zhao Z , Jia Q , Wu MS , et al. a Natural compound from solanum nigrum L., inhibits growth and metastasis of osteosarcoma through GSK3beta inactivation‐mediated repression of the hedgehog/Gli1 pathway. Clin Cancer Res. 2018;24(1):130‐144. 28951519 10.1158/1078-0432.CCR-17-0692

[13] Zhang R , Gilbert S , Yao X , et al. Natural compound methyl protodioscin protects against intestinal inflammation through modulation of intestinal immune responses. Pharmacol Res Perspectives. 2015;3(2):e00118. 10.1002/prp2.118 PMC4448980 26038694

[14] Dou YX , Zhou JT , Wang TT , et al. Self‐nanoemulsifying drug delivery system of bruceine D: a new approach for anti‐ulcerative colitis. Int J Nanomed. 2018;13:5887‐5907. 10.2147/IJN.S174146 PMC6167998 30319255

[15] Hall IH , Lee KH , Imakura Y , Okano M , Johnson A . Anti‐inflammatory agents III: Structure‐activity relationships of brusatol and related quassinoids. J Pharm Sci. 1983;72(11):1282‐1284. 6417321 10.1002/jps.2600721111

[16] Wright CW , Anderson MM , Allen D , et al. Quassinoids exhibit greater selectivity against plasmodium falciparum than against entamoeba histolytica, giardia intestinalis or toxoplasma gondii in vitro. J Eukaryot Microbiol. 1993;40(3):244‐246. 8508162 10.1111/j.1550-7408.1993.tb04910.x

[17] NoorShahida A , Wong TW , Choo CY . Hypoglycemic effect of quassinoids from Brucea javanica (L.) Merr (Simaroubaceae) seeds. J Ethnopharmacol. 2009;124(3):586‐591. 19439174 10.1016/j.jep.2009.04.058

[18] Lau ST , Lin ZX , Liao Y , Zhao M , Cheng CH , Leung PS . Bruceine D induces apoptosis in pancreatic adenocarcinoma cell line PANC‐1 through the activation of p38‐mitogen activated protein kinase. Cancer Lett. 2009;281(1):42‐52. 19286308 10.1016/j.canlet.2009.02.017

[19] Zhang JY , Lin MT , Tung HY , et al. Bruceine D induces apoptosis in human chronic myeloid leukemia K562 cells via mitochondrial pathway. Am J Cancer Res. 2016;6(4):819‐826. 27186433 PMC4859886

[20] Cheng Z , Yuan X , Qu Y , et al. Bruceine D inhibits hepatocellular carcinoma growth by targeting beta‐catenin/jagged1 pathways. Cancer Lett. 2017;403:195‐205. 28645563 10.1016/j.canlet.2017.06.014

[21] Peng Y , Wang Y , Tang N , et al. Andrographolide inhibits breast cancer through suppressing COX‐2 expression and angiogenesis via inactivation of p300 signaling and VEGF pathway. J Exp Clin Cancer Res. 2018;37(1):248. 30314513 10.1186/s13046-018-0926-9 PMC6186120

[22] Liu J , Chang B , Li Q , et al. Redox‐responsive dual drug delivery nanosystem suppresses cancer repopulation by abrogating doxorubicin‐promoted cancer stemness, metastasis, and drug resistance. Adv Sci. 2019;6(7):1801987. 10.1002/advs.201801987 PMC6446919 31139556

[23] Groner B , von Manstein V . Jak Stat signaling and cancer: opportunities, benefits and side effects of targeted inhibition. Mol Cell Endocrinol. 2017;451:1‐14. 28576744 10.1016/j.mce.2017.05.033

[24] Ryu K , Choy E , Yang C , et al. Activation of signal transducer and activator of transcription 3 (Stat3) pathway in osteosarcoma cells and overexpression of phosphorylated‐Stat3 correlates with poor prognosis. J Orthop Res. 2010;28(7):971‐978. 20063378 10.1002/jor.21088

[25] Wu J , Zhang J , Shen B , et al. Long noncoding RNA lncTCF7, induced by IL‐6/STAT3 transactivation, promotes hepatocellular carcinoma aggressiveness through epithelial‐mesenchymal transition. J Exp Clin Cancer Res. 2015;34:116. 26452542 10.1186/s13046-015-0229-3 PMC4600266

[26] Rhee YH , Jeong SJ , Lee HJ , et al. Inhibition of STAT3 signaling and induction of SHP1 mediate antiangiogenic and antitumor activities of ergosterol peroxide in U266 multiple myeloma cells. BMC Cancer. 2012;12:28. 22260501 10.1186/1471-2407-12-28 PMC3292511

[27] Huang TT , Su JC , Liu CY , Shiau CW , Chen KF . Alteration of SHP‐1/p‐STAT3 signaling: a potential target for anticancer therapy. Int J Mol Sci. 2017;18(6):1234. 10.3390/ijms18061234 PMC5486057 28594363

[28] Schust J , Sperl B , Hollis A , Mayer TU , Berg T . Stattic: a small‐molecule inhibitor of STAT3 activation and dimerization. Chem Biol. 2006;13(11):1235‐1242. 17114005 10.1016/j.chembiol.2006.09.018

[29] Pan X , Wang C , Li Y , Zhu L , Zhang T . Protective autophagy induced by physcion suppresses hepatocellular carcinoma cell metastasis by inactivating the JAK2/STAT3 Axis. Life Sci. 2018;214:124‐135. 30389439 10.1016/j.lfs.2018.10.064

[30] Lu J , Song G , Tang Q , et al. MiR‐26a inhibits stem cell‐like phenotype and tumor growth of osteosarcoma by targeting Jagged1. Oncogene. 2017;36(2):231‐241. 27270422 10.1038/onc.2016.194

[31] Liu W , Zhao Z , Wang Y , et al. Dioscin inhibits stem‐cell‐like properties and tumor growth of osteosarcoma through Akt/GSK3/beta‐catenin signaling pathway. Cell Death Dis. 2018;9(3):343. 29497056 10.1038/s41419-018-0363-x PMC5832770

[32] Martins‐Neves SR , Paiva‐Oliveira DI , Fontes‐Ribeiro C , Bovee J , Cleton‐Jansen AM , Gomes C . IWR‐1, a tankyrase inhibitor, attenuates Wnt/beta‐catenin signaling in cancer stem‐like cells and inhibits in vivo the growth of a subcutaneous human osteosarcoma xenograft. Cancer Lett. 2018;414:1‐15. 29126913 10.1016/j.canlet.2017.11.004

[33] Peng L , Jiang D . Resveratrol eliminates cancer stem cells of osteosarcoma by STAT3 pathway inhibition. PLoS ONE. 2018;13(10):e0205918. 30356255 10.1371/journal.pone.0205918 PMC6200233

[34] Wang L , Xue GB . Catalpol suppresses osteosarcoma cell proliferation through blocking epithelial‐mesenchymal transition (EMT) and inducing apoptosis. Biochem Biophys Res Commun. 2018;495(1):27‐34. 29032182 10.1016/j.bbrc.2017.10.054

[35] Tu Y . The discovery of artemisinin (qinghaosu) and gifts from Chinese medicine. Nat Med. 2011;17(10):1217‐1220. 21989013 10.1038/nm.2471

[36] Ma B , Zhu J , Zhao A , et al. Raddeanin A, a natural triterpenoid saponin compound, exerts anticancer effect on human osteosarcoma via the ROS/JNK and NF‐kappaB signal pathway. Toxicol Appl Pharmacol. 2018;353:87‐101. 29847772 10.1016/j.taap.2018.05.025

[37] Hanahan D , Weinberg RA . Hallmarks of cancer: the next generation. Cell. 2011;144(5):646‐674. 21376230 10.1016/j.cell.2011.02.013

[38] Ingham M , Schwartz GK . Cell‐cycle therapeutics come of age. J Clin Oncol. 2017;35(25):2949‐2959. 28580868 10.1200/JCO.2016.69.0032 PMC6075824

[39] Williams GH , Stoeber K . The cell cycle and cancer. J Pathol. 2012;226(2):352‐364. 21990031 10.1002/path.3022

[40] Wang Y , Deng X , Yu C , et al. Synergistic inhibitory effects of capsaicin combined with cisplatin on human osteosarcoma in culture and in xenografts. J Exp Clin Cancer Res. 2018;37(1):251. 30326933 10.1186/s13046-018-0922-0 PMC6192127

[41] Sun S , Xie F , Xu X , et al. Advanced oxidation protein products induce S‐phase arrest of hepatocytes via the ROS‐dependent, beta‐catenin‐CDK2‐mediated pathway. Redox Biol. 2018;14:338‐353. 29032312 10.1016/j.redox.2017.09.011 PMC5975226

[42] Lim S , Kaldis P . Cdks, cyclins and CKIs: roles beyond cell cycle regulation. Development. 2013;140(15):3079‐3093. 23861057 10.1242/dev.091744

[43] Li YG , Ji DF , Zhong S , et al. Polysaccharide from phellinus linteus induces S‐phase arrest in HepG2 cells by decreasing calreticulin expression and activating the P27kip1‐cyclin A/D1/E‐CDK2 pathway. J Ethnopharmacol. 2013;150(1):187‐195. 24001891 10.1016/j.jep.2013.08.028

[44] Zhang T , Li J , Yin F , et al. Toosendanin demonstrates promising antitumor efficacy in osteosarcoma by targeting STAT3. Oncogene. 2017;36(47):6627‐6639. 28783167 10.1038/onc.2017.270 PMC5702716

[45] Zhang T , Li S , Li J , et al. Natural product pectolinarigenin inhibits osteosarcoma growth and metastasis via SHP‐1‐mediated STAT3 signaling inhibition. Cell Death Dis. 2016;7(10):e2421. 27735939 10.1038/cddis.2016.305 PMC5133974

[46] Abarrategi A , Tornin J , Martinez‐Cruzado L , et al. Osteosarcoma: cells‐of‐origin. cancer stem cells, and targeted therapies. Stem Cells Int. 2016;2016:3631764. 27366153 10.1155/2016/3631764 PMC4913005

[47] Yan GN , Lv YF , Guo QN . Advances in osteosarcoma stem cell research and opportunities for novel therapeutic targets. Cancer Lett. 2016;370(2):268‐274. 26571463 10.1016/j.canlet.2015.11.003

[48] Brown HK , Tellez‐Gabriel M , Heymann D . Cancer stem cells in osteosarcoma. Cancer Lett. 2017;386:189‐195. 27894960 10.1016/j.canlet.2016.11.019

[49] Martins‐Neves SR , Lopes AO , de Carmo A , et al. Therapeutic implications of an enriched cancer stem‐like cell population in a human osteosarcoma cell line. BMC Cancer. 2012;12:139. 22475227 10.1186/1471-2407-12-139 PMC3351999

[50] Rios‐Fuller TJ , Ortiz‐Soto G , Lacourt‐Ventura M , et al. Ganoderma lucidum extract (GLE) impairs breast cancer stem cells by targeting the STAT3 pathway. Oncotarget. 2018;9(89):35907‐35921. 30542507 10.18632/oncotarget.26294 PMC6267592

[51] Liu Y , Choi DS , Sheng J , et al. HN1L promotes triple‐negative breast cancer stem cells through LEPR‐STAT3 pathway. Stem Cell Rep. 2018;10(1):212‐227. 10.1016/j.stemcr.2017.11.010 PMC5768915 29249663

